# Preliminary experience of single operative port thoracoscopic anatomical lesion resection for congenital lung malformations in segments S9-10

**DOI:** 10.3389/fped.2025.1649456

**Published:** 2025-09-26

**Authors:** Rui Guo, Yunpeng Zhai, Huashan Zhao, Hongxiu Xu, Gang Shen, Sai Huang, Shisong Zhang

**Affiliations:** ^1^ Department of Thoracic and Tumor Surgery, Children’s Hospital Affiliated to Shandong University, Jinan, Shandong, China; ^2^ Department of Thoracic and Tumor Surgery, Jinan Children’s Hospital, Jinan, Shandong, China

**Keywords:** congenital lung malformations, thoracoscopic anatomical lesion resection, singleoperative port, pulmonary segments S9-10, multi-portal video-assisted thoracic surgery

## Abstract

**Purpose:**

To compare the clinical outcomes of single operative port thoracoscopic anatomical lesion resection (TALR) with multi-portal video-assisted thoracic surgery (M-VATS) in the treatment of congenital lung malformations (CLMs) located in pulmonary segments S9-10, and to evaluate its safety and feasibility in routine practice.

**Methods:**

We retrospectively analysed 46 paediatric CLMs cases treated thoracoscopically at our institution from January 2023 to January 2025. Patients were grouped by surgical approach: 13 underwent single operative port TALR, and 33 underwent M-VATS. Clinical parameters were compared between groups.

**Results:**

Compared to M-VATS, the single operative port TALR showed significantly reduced blood loss (*P* = 0.01), chest tube time (*P* = 0.04), hospital stays (*P* = 0.04), and incision length (*P* < 0.01). No bronchopleural fistulas, conversions to open surgery, no recurrence or residual lesions occurred in either group.

**Conclusion:**

single operative port TALR is safe, feasible, minimally invasive, and offers excellent cosmetic outcomes, representing a promising surgical technique for treating CLMs involving segments S9-10.

## 1 Introduction

Congenital lung malformations (CLMs), including congenital pulmonary airway malformation (CPAM) and pulmonary sequestration (PS), represent rare developmental anomalies arising during embryogenesis (1). Although typically benign, CLMs can predispose patients to recurrent infections, respiratory compromise, and potential malignant transformation (2, 3). Therefore, early surgical intervention is strongly recommended to prevent these adverse outcomes. Recent advancements in minimally invasive surgical techniques have positioned thoracoscopic pulmonary segmentectomy as the preferred surgical approach for CLMs. However, segmental resection of the posterior basal segments (S9–10) remains technically challenging, given their deep anatomical location, adjacency to multiple pulmonary segments, and complex vascular anatomy (4, 5). To address these challenges, thoracoscopic anatomical lesion resection (TALR) has emerged as a promising method. The central principle of TALR involves the precise identification and dissection of internal and external lesion boundaries; specifically, the external boundary demarcates the interface between pathological and healthy lung tissue, while the internal boundary typically aligns with intersegmental veins (6, 7). Despite its clinical advantages, conventional TALR typically employs a three-port thoracoscopic technique, involving multiple incisions. This multi-incision approach increases the risk of intercostal nerve injury, resulting in heightened postoperative pain and negatively affecting cosmetic outcomes (8, 9). Recognizing that TALR procedures involve limited instrument manipulation confined primarily to the lesion area, we propose an innovative single operative port approach. By consolidating two operative ports into a single intercostal incision, we aim to minimize chest wall trauma and enhance cosmetic outcomes without compromising surgical efficacy. To our knowledge, no prior studies have evaluated this single operative port TALR approach specifically for posterior basal segments (S9–10).

This retrospective study collected clinical data on patients with CLMs who underwent either single operative port TALR or multi-portal video-assisted thoracic surgery (M-VATS) at our institution. The objective was to compare the clinical advantages and limitations of these two approaches and to assess the safety and feasibility of the single operative port TALR in routine surgical practice.

## 2 Methods

### 2.1 Study design and patients

The Ethics Committee of Jinan Children's Hospital (Children's Hospital Affiliated to Shandong University) approved this study (the ethical approval number: SDFE-IRB/P-2023024) and followed the principles of the Declaration of Helsinki. The parents of each child provided written informed consent.

In our hospital, between January 2025 and January 2023, 46 children diagnosed with CLMs underwent surgical treatment. Preoperative assessments included enhanced chest CT scans to confirm the diagnosis, assess vascular abnormalities (including their location, quantity, and size), and formulate a surgical plan. Data on the patients' basic information, CLMs characteristics (such as lesion type and location), and perioperative details (such as operation duration, blood loss, drainage duration, complications, and postoperative hospital stay) were all documented. The inclusion criteria were as follows: (1) patients diagnosed with CPAM or ILS; (2) lesions confined to unilateral S9-10 with no acute inflammatory response; (3) lesion diameter of ≥2 cm (10). Exclusion criteria involved: children with associated conditions impacting cardiopulmonary function, such as congenital heart disease, restrictive or obstructive chest wall diseases, or other abnormalities requiring simultaneous surgical intervention.

### 2.2 Surgical technique

#### 2.2.1 Single operative port thoracoscopic anatomical lesion resection

**Figure 1:**
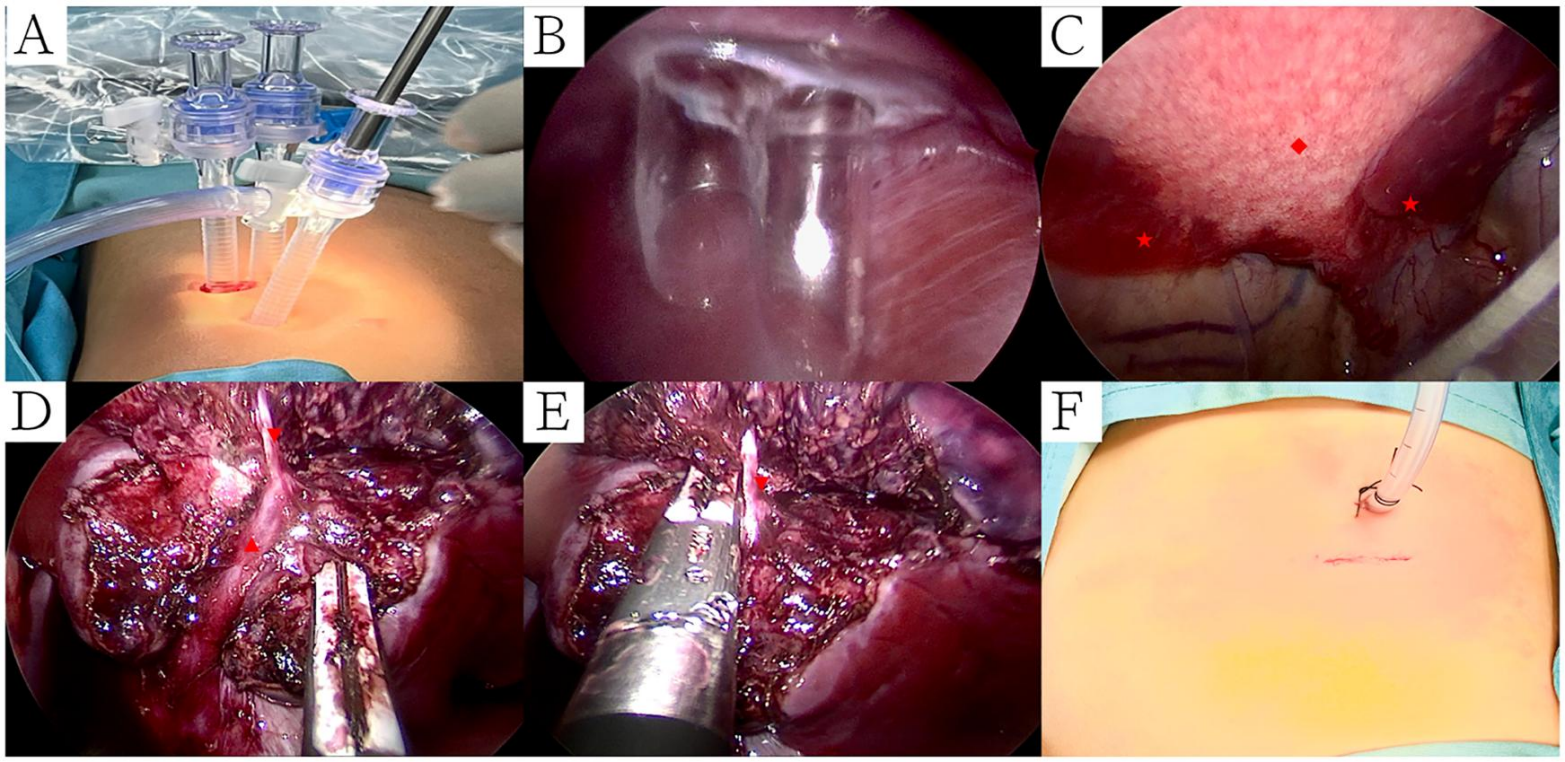
Surgical procedure of single operative port TALR. **(A)** A 0.5-cm incision at the 7th intercostal space along the scapular line was created for placement of an observational Trocar. Additionally, a single 1.2-cm transverse incision at the 8th intercostal space along the scapular line accommodated two parallel 5-mm Trocars, forming a single operative port; **(B)** Two Trocars staggered insertion depths to avoid instrument interference within the thoracic cavity; **(C)** An electric hook was employed to delineate the external boundaries of the lesion precisely; **(D)** Intersegmental veins demarcating the internal boundary between healthy and affected lung tissue were preserved; **(E)** Lung tissue between these established internal (intersegmental veins) and external (lesion margins) boundaries was precisely transected and sealed using LigaSure™; **(F)** Surgical incision was sutured. Symbols: CPAM: ◆, Normal Lungs: ★, Intersegmental veins: ▴, Segmental veins: ▾.

**Figure 2:**
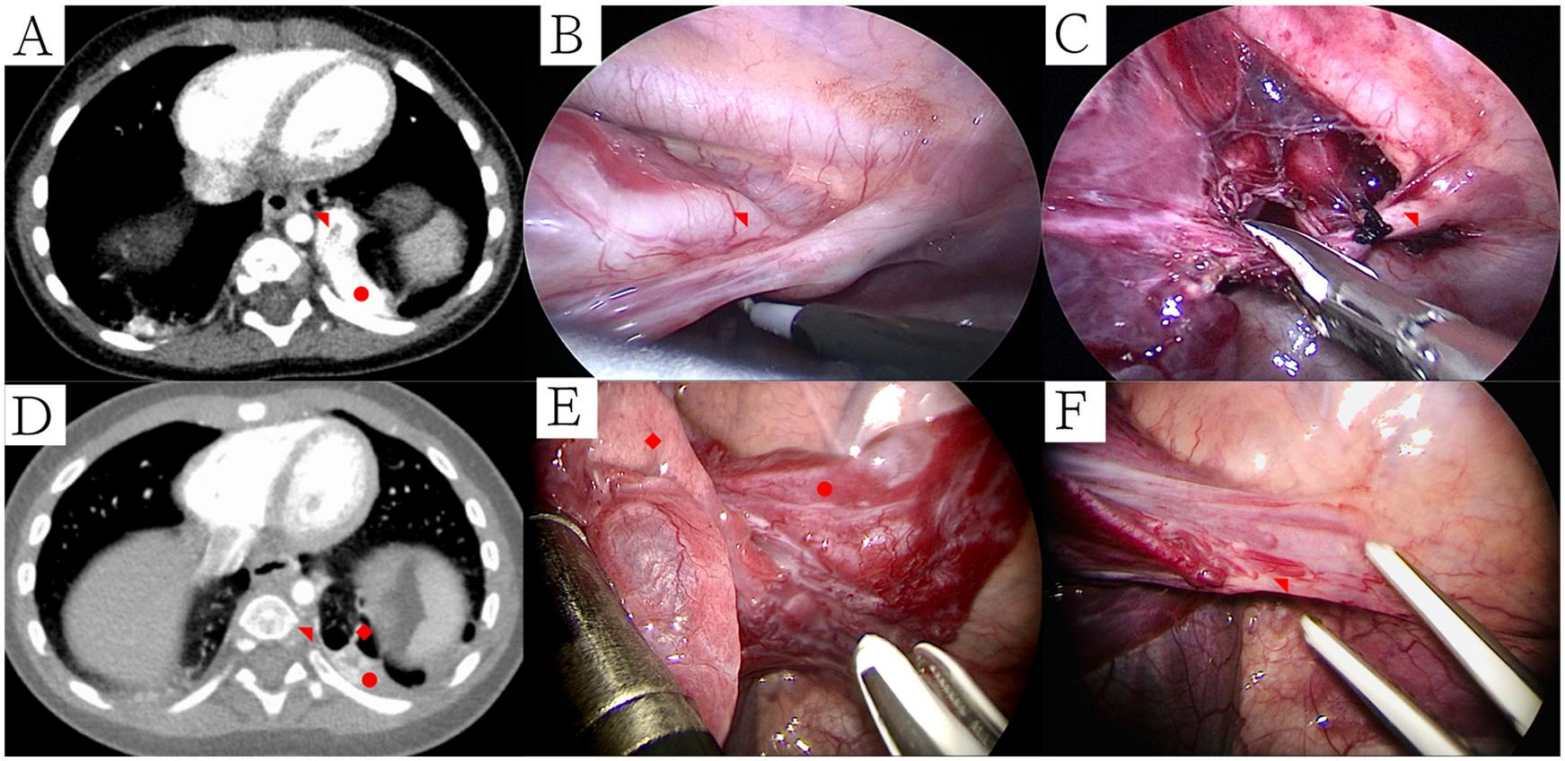
Ct and thoracoscopic manifestations of PS and treatment of abnormal blood vessels. **(A)** PS supplied by larger abnormal blood vessels (mediastinal window); **(B)** Electric hook freed larger abnormal blood vessels; **(C)** Larger abnormal blood vessels were proximally ligated prior to division with LigaSure™; **(D)** CPAM with PS supplied by smaller abnormal blood vessels (mediastinal window); **(E)** Thoracoscopic manifestations of CPAM with ILS; **(F)** Smaller abnormal blood vessels were sealed and transected using LigaSure™. Symbols: CPAM: ◆, Pulmonary sequestration: ●, Abnormal blood artery: ◥.

**Figure 3:**
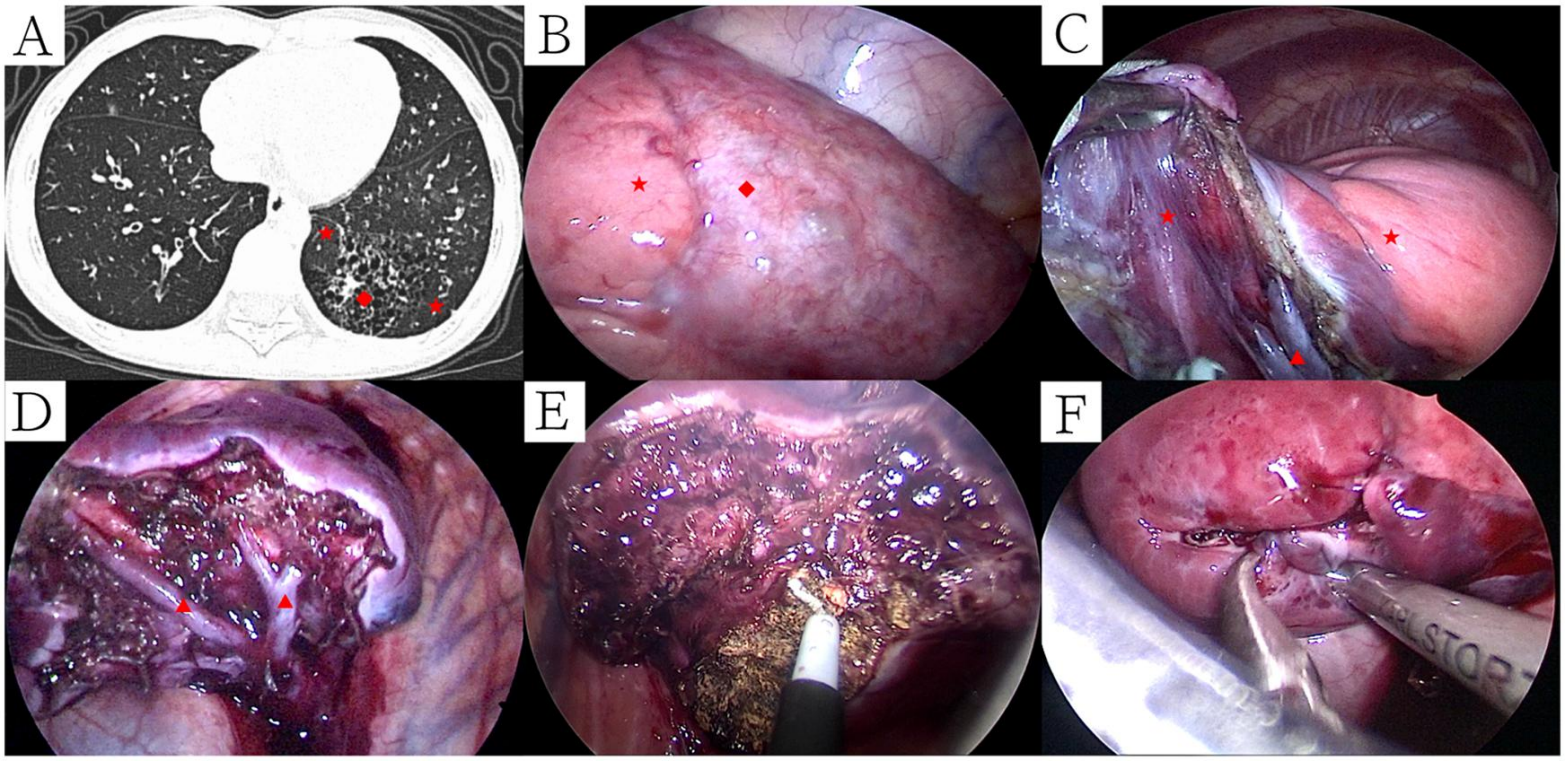
Ct manifestations and intraoperative management of CPAM patients with recurrent pulmonary infections recently. **(A)** CT manifestations of CPAM patients with recurrent pulmonary infections recently (lung window); **(B)** External boundary between healthy and affected lung tissue; **(C)** Cut and separate along the boundary between CPAM and normal lung tissue; **(D)** Internal boundary after removal of CPAM (segmental veins); **(E)** Identified bleeding sites were meticulously cauterized with the electric hook; **(F)** Resection margins were reinforced using continuous 5-0 PDSII sutures. Symbols: CPAM: ◆, Normal Lungs: ★, Intersegmental vein: ▴.

All surgical procedures were conducted under general anesthesia. Single-lumen endotracheal intubation with selective bronchial occlusion of the affected main bronchus was performed to achieve unilateral lung ventilation. Patients were placed in the lateral decubitus position. A 0.5-cm incision at the 7th intercostal space along the scapular line was created for placement of an observational Trocar. Additionally, a single 1.2-cm transverse incision at the 8th intercostal space along the scapular line accommodated two parallel 5-mm Trocars, forming a single operative port with staggered insertion depths to avoid instrument interference within the thoracic cavity (Figures 1A,B). An artificial pneumothorax was established, maintaining a pressure of 4–8 mmHg (1 mmHg = 0.133 kPa) and a flow rate of 2–4 L/min. Operative forceps gently manipulated the affected lung to facilitate optimal collapse. An electric hook was employed to delineate the external boundaries of the lesion precisely (Figure 1C). The inferior pulmonary ligament was carefully dissected and transected in layers. In cases of PS, aberrant feeding arteries with diameters exceeding 2 mm were proximally ligated prior to division with LigaSure™ (Figures 2A–C). Arteries measuring 2 mm or smaller were directly sealed and transected using LigaSure™ (Figures 2D–F). The lower pulmonary veins and their branches were meticulously dissected with the electric hook. Segmental veins supplying the lesion were ligated and divided, while intersegmental veins demarcating the internal boundary between healthy and affected lung tissue were preserved (Figures 1D,E). Lung tissue between these established internal (intersegmental veins) and external (lesion margins) boundaries was precisely transected and sealed using LigaSure™. The excised lung specimens were removed from the thoracic cavity using a retrieval bag. The cavity was thoroughly irrigated with warmed saline solution. Lung surfaces and resection margins were carefully inspected for active bleeding or air leaks. Identified bleeding sites were meticulously cauterized with the electric hook, and the resection margins were reinforced using continuous 5-0 PDSII sutures (Figures 3E,F). After confirming satisfactory lung re-expansion, a closed thoracic drainage tube was inserted through the observational Trocar incision at the 7th intercostal space (Figure 1F). The chest tube was subsequently removed when air leakage ceased, and daily drainage volume was consistently below 20 ml.

#### 2.2.2 Multi-portal video-assisted thoracic surgery

The observation port was placed at the 8th intercostal space along the midaxillary line, while two working ports were established at the 8th intercostal space on the anterior axillary line and the 7th intercostal space on the subscapular line. Using an electric hook, the external margin of the lesion was first delineated. The lower pulmonary ligament was divided to expose the inferior pulmonary vein, and both the vein and its branches (V6 and the common basal vein, CBV) were carefully mobilized with the electric hook. The parenchyma between V6 and the outer border of its corresponding lesion was then transected and sealed using a LigaSure™ device along the S9-10-adjacent side of V6. Following this, the CBV and its branches were dissected in accordance with the venous anatomy identified on contrast-enhanced chest CT. The intra-segmental vein draining the lesion was divided, whereas the intra-segmental vein separating the lesion from normal lung parenchyma was preserved to serve as the internal boundary. Lung tissue between this internal venous landmark and the external margin was subsequently transected and sealed with LigaSure™. The next step involved locating the bronchus and artery supplying the lesion, which were individually isolated, ligated, and divided. All cut surfaces of vessels, bronchus, and lung parenchyma were carefully inspected; once the absence of air leaks or bleeding was confirmed, closure was reinforced with 5-0 Prolene sutures. After re-expansion of the lung, a closed thoracic drainage tube was inserted through the anterior axillary line port at the 8th intercostal space.

### 2.3 Statistical analysis

Statistical analyses were performed using SPSS version 26.0. Continuous variables were expressed as either the mean ± standard deviation or the median, depending on the data distribution. Normality was assessed prior to comparative analysis. For normally distributed data, comparisons between groups were conducted using the independent samples t-test; for non-normally distributed data, the Mann–Whitney U test (rank-sum test) was applied. Categorical variables were presented as frequencies and percentages, and comparisons were made using the Pearson chi-square test or Fisher's exact test, as appropriate. A two-sided *P* value of <0.05 was considered statistically significant.

## 3 Results

The characteristics of this study population are presented in Table 1. Analyses of the single operative port TALR and M-VATS groups were conducted using univariate analysis (Table 1). Intraoperative and postoperative variables are shown in Table 2.

**Table 1:** Baseline characteristics of the study population and univariate comparison of single operative port group vs. M-VATS group.

Characteristics	Single operative port group (*n* = 13)	M-VATS group (*n* = 33)	*P*
Age (month)	11.8 (3.5–84)	23.9 (3–96)	0.13
Weight (kg)	9.0 (5.1–21.6)	12.2 (6.4–33)	0.10
Male	7 (53.8%)	17 (51.5%)	0.89
Female	6 (46.2%)	16 (48.5%)	
CPAM	10 (76.1%)	26 (78.8%)	
CPAM + ILS	2 (15.4%)	4 (12.1%)	
ILS	1 (8.5%)	3 (9.1%)	

**Table 2:** Intraoperative and postoperative variables.

Variables	Single operative port group	M-VATS group	*P*
Operative time (min)	69.9 (25–101)	76.1 (33–118)	0.4
Blood loss (ml)	4.1 (2–10)	6.8 (3–20)	0.01
Chest tube time (days)	3.5 (3–5)	4.3 (3–8)	0.04
Duration of post-operative hospital stays (days)	5.6 (5–8)	6.5 (5–10)	0.04
Incision length (cm)	1.7 (1.6–1.9)	2.2 (2.1–2.5)	<0.01

There were no statistically significant differences in age (*P* = 0.15), weight (*P* = 0.20) and sex (*P* = 0.57) in the two groups (Table 1). In the univariate analysis, there were statistical significances between two groups in terms of reduced blood loss (*P* = 0.01), chest tube time (*P* = 0.04), hospital stays (*P* = 0.04), and incision length (*P* < 0.01).

The single operative port TALR group comprised 13 pediatric patients (7 males and 6 females), with ages ranging from 3.5 to 84.0 months (median, 4.8 months). Body weights ranged from 5.1 to 21.6 kg, with a median of 8.3 kg. CLMs were prenatally diagnosed via ultrasonography in 11 patients, with subsequent confirmation by chest computed tomography (CT) within one month after birth. The remaining two patients underwent CT scans following recurrent respiratory infections. Lesions were evenly distributed, with seven cases in the left lung (segments S9–10) and six in the right lung (segments S9–10). The largest lesion diameter ranged from 2.5 to 6.1 cm, with a median of 4.2 cm. Pathological classifications included ten cases of CPAM, one case of intralobar sequestration (ILS), and two combined cases of CPAM and ILS. Surgery was indicated by clinical respiratory symptoms in five patients (cough in three cases, asthma in two), while elective procedures were performed in eight asymptomatic patients upon guardian preference.

All surgical procedures were successfully completed without conversion to open thoracotomy. The median operative time was 68 min (range, 25–101 min). Median intraoperative blood loss was minimal at 3 ml (range, 2–10 ml). Chest tubes remained in place postoperatively for a median of 3 days (range, 3–5 days), and patients were hospitalized postoperatively for a median of 5 days (range, 5–8 days). No surgical mortality or major intraoperative complications occurred. Two patients developed postoperative subcutaneous emphysema, which resolved spontaneously with conservative management, without progression to pneumothorax or bronchopleural fistula. One patient, aged 84 months with a history of recurrent pneumonia, experienced sudden onset of dyspnea seven days post-discharge after a severe coughing episode. Chest radiography revealed a left-sided pneumothorax, managed effectively with closed thoracic drainage for seven days. Despite normalization of inflammatory biomarkers one month prior to surgery, CT scans indicated persistent inflammation within the lesion region (Figure 3A). Due to clear demarcation between the lesion and normal pulmonary tissue without visible bronchial involvement, the surgical margin was not sutured initially (Figures 3B–D). The subsequent pneumothorax recurrence was likely attributable to alveolar rupture secondary to fragile tissue persisting from chronic inflammation. Consequently, subsequent procedures have included meticulous cauterization and suture reinforcement of surgical margins to mitigate the risk of postoperative air leaks (Table 3).

**Table 3:** Clinical data of children undergoing single operative port TALR for CLMs in segments S9-10.

Patient number	Gender	Age (months)	Weight (kg)	Systematic (Y/N)	Location (L/R)	Operative time (min)	Blood loss (ml)	Drainage duration (days)	Duration of post-operative hospital stay (days)	Diagnosis	Complications	Follow-up time (months)
1	M	4.4	7.5	N	L	45	5	3	5	CPAM	N	3
2	F	3.5	5.1	Y	R	76	5	3	5	CPAM	N	3
3	F	8.9	8.5	Y	L	101	10	3	5	CPAM + ILS	N	3
4	F	9.8	10	N	L	73	5	3	6	CPAM + ILS	N	3
5	M	4.7	8.3	N	R	68	2	4	5	CPAM	subcutaneous emphysema	4
6	M	4.8	8.9	N	L	52	2	5	8	CPAM	N	4
7	M	6.8	8.5	N	R	54	3	3	5	ILS	N	6
8	F	5.7	8.7	N	L	57	3	3	6	CPAM	N	7
9	F	84	21.6	Y	L	74	3	3	5	CPAM	delayed pneumothorax	8
10	M	4.2	6.6	Y	L	97	3	4	6	CPAM	N	8
11	M	5.8	7.7	N	R	92	5	4	6	CPAM	N	8
12	M	6.6	8.5	N	R	25	2	4	5	CPAM	N	9
13	F	4.4	6.5	Y	R	95	5	4	6	CPAM	subcutaneous emphysema	12

All patients completed follow-up examinations at 3 months, 6 months, and 1 year postoperatively, with a median follow-up duration of 4 months (range, 3–12 months). No cases of hemoptysis or residual lesions were detected on follow-up chest CT scans.

## 4 Discussion

Congenital lung malformations (CLMs) are developmental anomalies of the lung or airway occurring during embryogenesis, typically presenting as localized structural abnormalities of pulmonary tissue or aberrant airway branching. The estimated incidence of CLMs ranges from 1 to 4.2 per 10,000 births (11). While CLMs are predominantly benign, they can predispose patients to recurrent infections, respiratory compromise, and long-term malignant transformation, notably bronchioloalveolar carcinoma (12). Consequently, early surgical intervention is recommended for symptomatic patients or those with lesion diameters exceeding 2 cm on imaging (13). The optimal surgical window is between 3 and 12 months post-birth, when cardiopulmonary stability and adequate thoracoscopic working space are ensured (14, 15). Thoracoscopic segmentectomy is currently the primary surgical technique for lesions located in segments S9–10. However, anatomical complexities inherent to these segments pose significant surgical challenges. Firstly, the deeply situated segmental hilum necessitates extensive dissection of segmental arteries (A9–10), bronchi (B9–10), and inferior pulmonary veins along with their branches (V6–10), thereby increasing the risk of injury to adjacent segments, notably S6 and S7–8. Secondly, intersegmental planes between segments S9–10 and segments S6 and S7–8 are often incompletely developed, complicating the accurate determination of surgical boundaries via traditional inflation-deflation methods, thus increasing the risk of incomplete resection or excessive normal tissue removal (5, 16). To overcome these difficulties, two alternative techniques have recently been proposed: modified thoracoscopic wedge resection and thoracoscopic anatomical lesion resection (TALR). Modified wedge resection leverages preoperative CT imaging to delineate the intersegmental venous anatomy, permitting targeted excision of lesions limited to segmental or subsegmental regions (17). Conversely, TALR is predicated on the theory of “duplicate malformations” in CLMs and emphasizes precise identification of both external boundaries (indicated by differences in lung collapse and vascular discoloration) and internal boundaries (marked by intersegmental veins), facilitating accurate lesion removal while preserving maximum healthy lung tissue (6). Despite their differing theoretical bases, both techniques share the goal of precise lesion boundary identification to achieve minimally invasive yet maximal tissue-conserving surgery. In this study, we employed preoperative CT to determine internal lesion boundaries based on intersegmental venous anatomy, while external boundaries were defined intraoperatively by cyst margins, congestion lines, lung collapse differences, and vascular color variations. Following ligation of segmental veins entering lesions, lung tissue between established internal and external boundaries was precisely divided using LigaSure™.

Conventionally, M-VATS comprising an observation port placed at the midaxillary line (7th–8th intercostal space) and two operative ports located along anterior and posterior axillary lines. Although this approach provides good surgical exposure and adequate workspace, multiple incisions elevate cumulative risks of intercostal nerve damage, increasing postoperative pain compared with single operative port methods (9). Additionally, dispersed incisions negatively impact cosmetic outcomes, potentially causing psychological distress for pediatric patients' families. Considering the limited instrument maneuvering required in TALR, we developed a single operative port thoracoscopic technique, consolidating two traditionally separate operative ports into one incision at the 8th intercostal space along the subscapular line. This strategy is theoretically advantageous due to limited anatomical exposure required, absence of extensive lung retraction, and relatively fixed intersegmental vein dissection pathways, minimizing instrument interference. Nevertheless, a major challenge of single operative port surgery is instrument collision. We overcame this difficulty using two key innovations: first, leveraging the natural curvature of pediatric intercostal spaces to separate instruments effectively; second, employing staggered-depth insertion of two parallel Trocars into the same intercostal space. This approach ensures distinct operative pathways, particularly beneficial when managing deep-seated or substantial aberrant vessels associated with pulmonary sequestration (PS). None of our 13 patients experienced visual obstruction due to inadequate instrument angles, and intraoperative management of aberrant vessels proceeded uneventfully without bleeding complications. When compared to M-VATS performed at our institution, the single operative port TALR also resulted in reduced blood loss (4.1 ml vs. 6.8 ml), shorter chest tube time (3.5 ml vs. 4.3 ml), shorter postoperative hospital stays (5.6 days vs. 6.5 days) and smaller incision length (1.7 ml vs. 2.2 ml), with all differences reaching statistical significance (*P* < 0.05).

Through precise delineation of lesion boundaries, single operative port TALR significantly reduced the potential for residual lesion occurrence, with no such cases documented in our series. However, because single operative port TALR is technically challenging and does not strictly adhere to anatomical segmentectomy standards, potential complications—such as increased intraoperative bleeding or postoperative air leaks—remain a concern. These complications could adversely impact surgical outcomes, extend hospitalization duration, or necessitate additional interventions (18, 19). To mitigate bleeding risk during CLMs resection, we initially dissected intersegmental veins defining internal boundaries and sequentially separated segmental veins along their adventitia using LigaSure™, reducing blood loss associated with blunt dissection. Given that aberrant vessels in PS vary widely in size and are frequently obscured within the inferior pulmonary ligament, meticulous preoperative assessment of CT scans to determine their size and distribution is essential. Intraoperatively, larger vessels (>2 mm diameter) were proximally ligated before division, whereas smaller vessels (≤2 mm) were directly sealed and divided using LigaSure™. Using meticulous preoperative planning and careful intraoperative management, intraoperative blood loss remained minimal (range, 2–10 ml) without any cases necessitating transfusion. Postoperative air leaks represent a persistent complication in thoracoscopic lung resection, particularly among patients with histories of recurrent pneumonia (20). In our study, three patients developed postoperative air leaks—two presenting as self-resolving subcutaneous emphysema, and one developing pneumothorax requiring intervention 7 days post-discharge following vigorous coughing. The latter case involved an 84-month-old patient with a history of recurrent pneumonia, in whom persistent inflammation was intraoperatively evident despite normalized inflammatory markers. Initially, surgical margins were not sutured as bronchial structures were not visibly involved; however, the pneumothorax recurrence was attributed to fragile inflammatory-stage tissue ruptured by vigorous coughing. Thus, we have subsequently implemented rigorous preventive measures, including ensuring complete inflammation resolution on CT scans at least three months post-infection and meticulous intraoperative margin reinforcement by extensive cauterization and continuous 5-0 PDSII sutures.

This study is limited by its retrospective nature, small sample size, and short-term follow-up, which preclude definitive evaluation of long-term functional outcomes such as spirometry, exercise tolerance, or ventilation/perfusion recovery. Moreover, the absence of standardized assessments for postoperative pain and cosmesis restricts comprehensive appraisal of patient-centered benefits. Finally, the technical demands of single operative port TALR may extend the learning curve. Future multicenter prospective studies with larger cohorts and validated outcome measures are required to establish the long-term safety, efficacy, and quality-of-life advantages of this approach.

In conclusion, Single operative port TALR offers significant advantages, including minimal invasiveness, rapid postoperative recovery, and superior cosmetic outcomes. The described technique effectively resolves instrument interference and postoperative air leaks, demonstrating excellent short-term efficacy for managing congenital lung malformations in pulmonary segments S9–10. This method is clinically feasible, providing a valuable surgical alternative for pediatric CLMs treatment.

## Data Availability

The original contributions presented in the study are included in the article/Supplementary Material, further inquiries can be directed to the corresponding author.
